# Supplementary figures and images for: Transcriptome Profiling Identifies Candidate Genes Contributing to Male and Female Gamete Development in Synthetic Brassica Allohexaploids
Source: Plants (Basel). 2022 Jun 13;11(12):1556. doi: 10.3390/plants11121556 (PMC9228180; doi:10.3390/plants11121556)

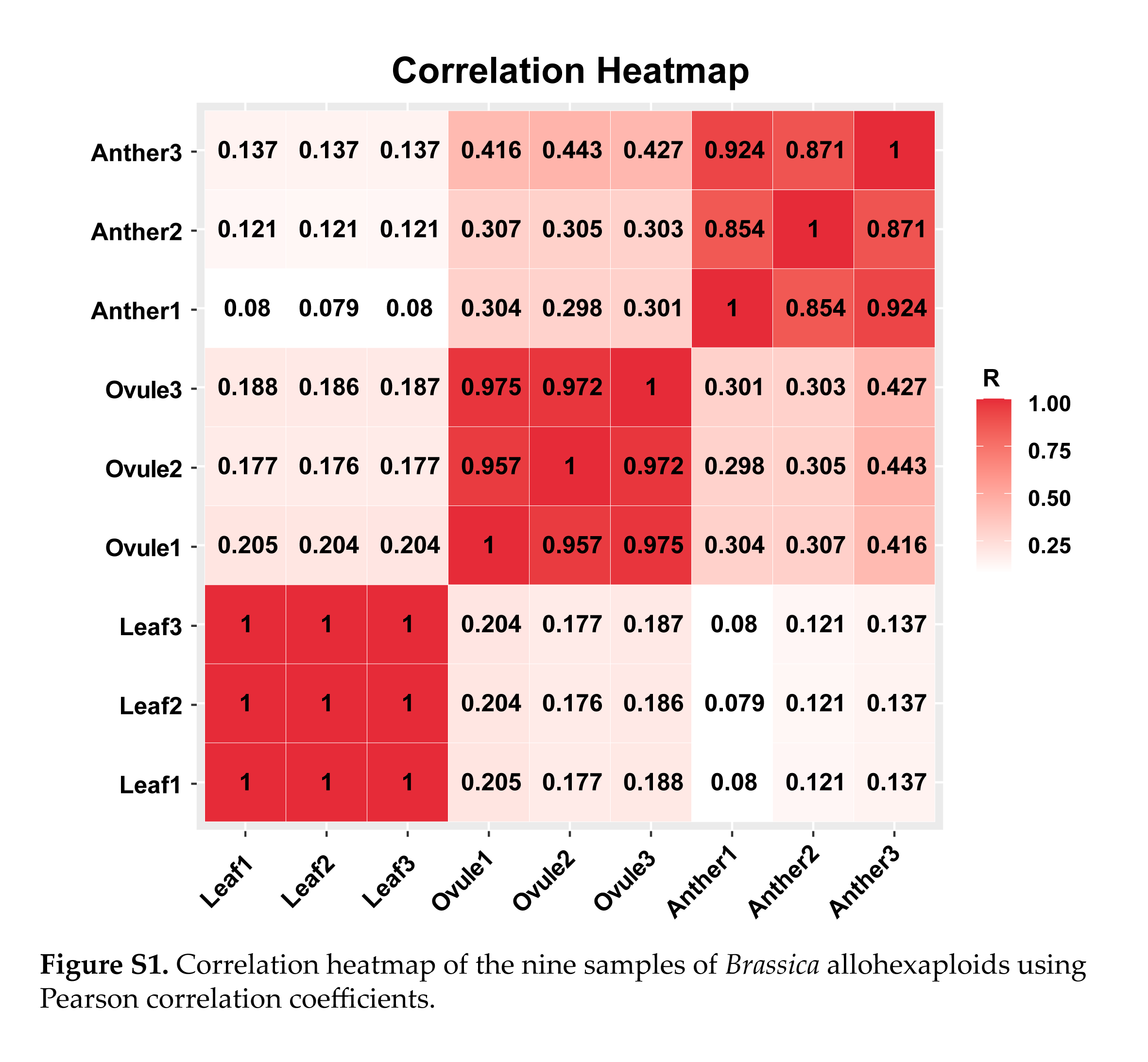

Supplement: Supplementary file 1 [file plants-11-01556-s001.zip › Figure S1.tif]

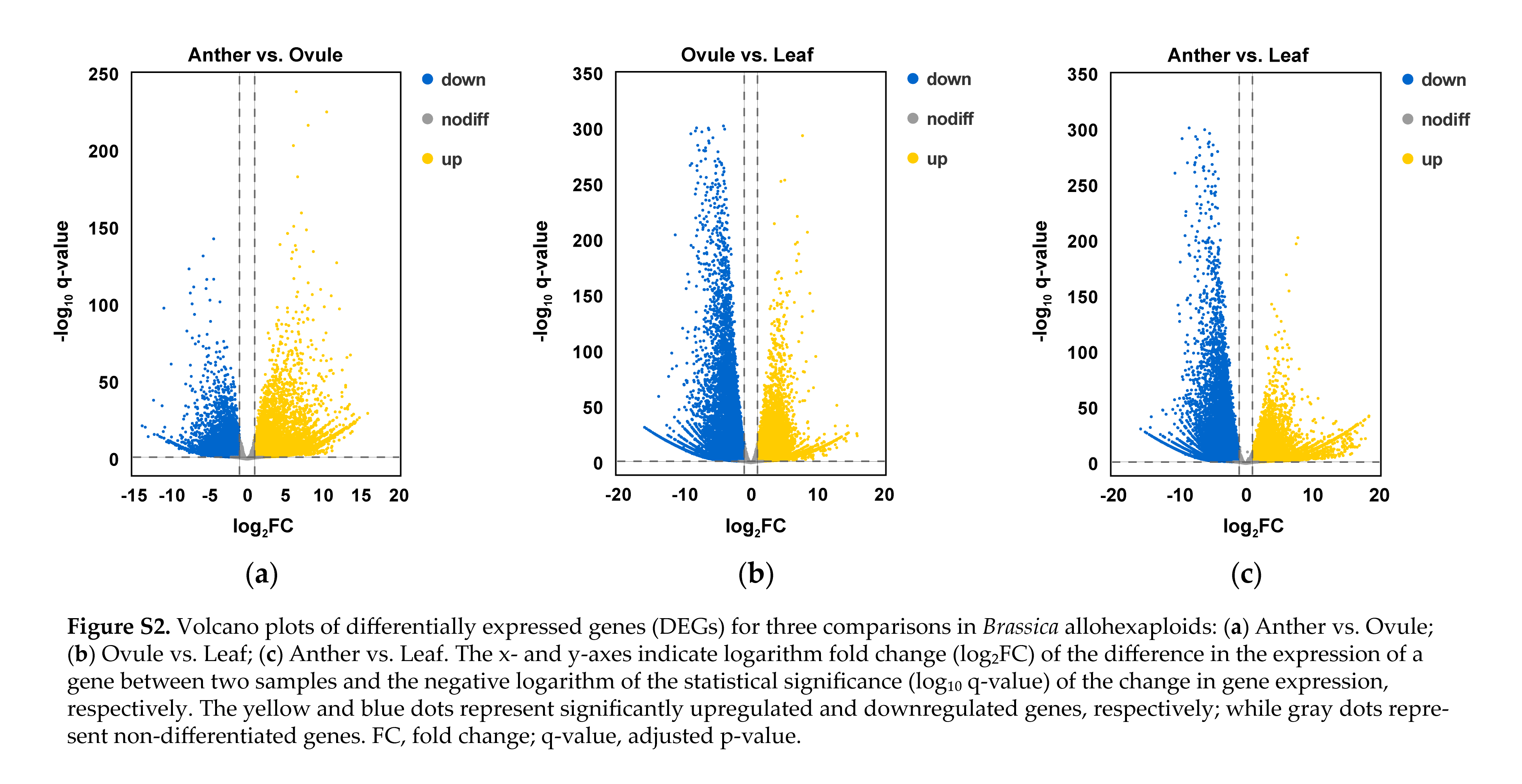

Supplement: Supplementary file 1 [file plants-11-01556-s001.zip › Figure S2.tif]
